# Supplementary material for: Synthesis and Biological Evaluation of Novel 2-Amino-1,4-Naphthoquinone Amide-Oxime Derivatives as Potent IDO1/STAT3 Dual Inhibitors with Prospective Antitumor Effects
Source: Molecules. 2023 Aug 19;28(16):6135. doi: 10.3390/molecules28166135 (PMC10459814; doi:10.3390/molecules28166135)
Supplement: Supplementary file 1 [file molecules-28-06135-s001.zip › molecules-2412493-supplementary.pdf]

Supplementary Materials

Synthesis and Biological Evaluation of Novel 2-Amino-1,4-Naphthoquinone Amide-Oxime Derivatives as Potent IDO1/STAT3 Dual Inhibitors with Prospective Antitumor Effects

Ri-Zhen Huang [1, †], Qiao-Ling Liang [1,†], Xiao-Teng Jing [2,3], Ke Wang [1], Hui-Yong Zhang [1], Heng-Shan Wang [2], Xian-Li Ma [1,*], Jian-Hua Wei [1,*], and Ye Zhang [1,*]

1 Guangxi Key Laboratory of Drug Discovery and Optimization, Guangxi Engineering Research Center for Pharmaceutical Molecular Screening and Druggability Evaluation, School of Pharmacy, Guilin Medical Univer-sity, Guilin 541199, China
2 State Key Laboratory for the Chemistry and Molecular Engineering of Medicinal Resources, Collaborative Innovation Center for Guangxi Ethnic Medicine, School of Chemistry and Pharmaceutical Sciences of Guangxi Normal University, Guilin, 541004, China
3 Department of Chemistry & Pharmaceutical Science, Guilin Normal College, Xinyi Road 15, Guangxi 541001, China

[*]Corresponding author.

[*]Corresponding author. Guangxi Key Laboratory of Drug Discovery and Optimization, Guangxi Engineering Research Center for Pharmaceutical Molecular Screening and Druggability Evaluation, School of Pharmacy, Guilin Medical University, Guilin 541199, China

E-mail addresses: mxl78@glmc.edu.cn (X. -L. M.), weijh1124@glmc.edu.cn (J.-H. W.), zhangye81@126.com (Y. Z.).

[†]Co-first author: These authors contributed equally to this work.

^{1}H NMR, ^{13}C NMR and HR-MS of compounds **7a−7c** and **NK1-NK3**:

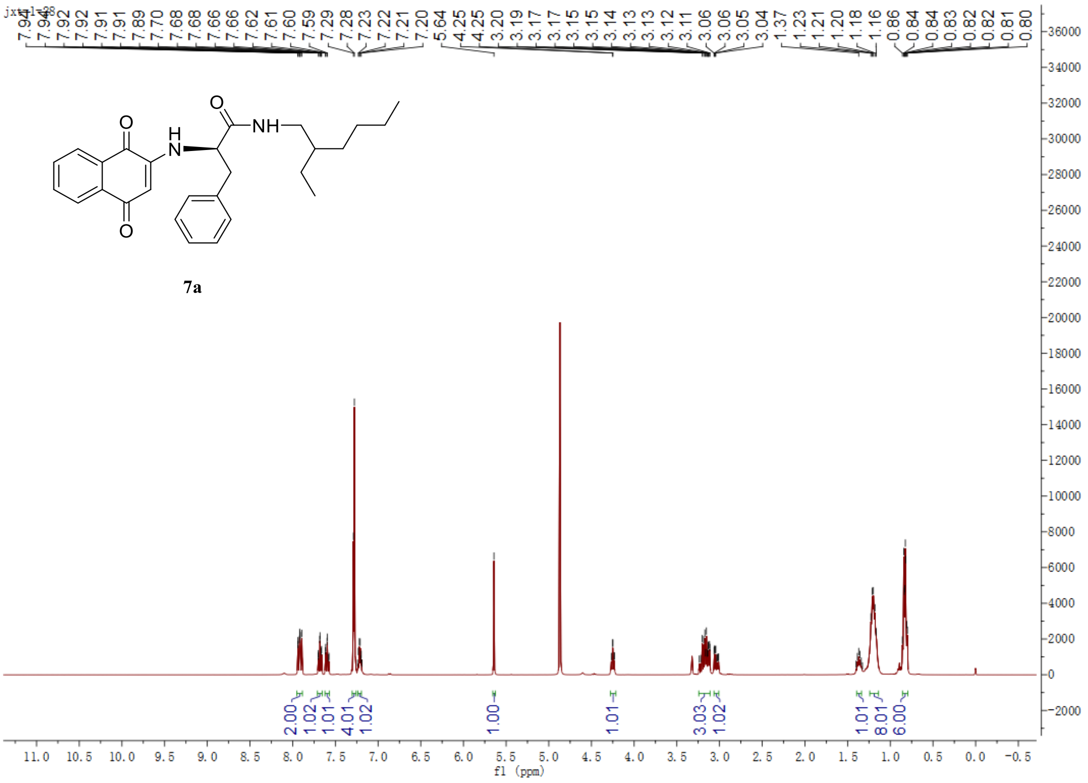

Figure S1. ^{1}H NMR Spectrum of compound **7a**.

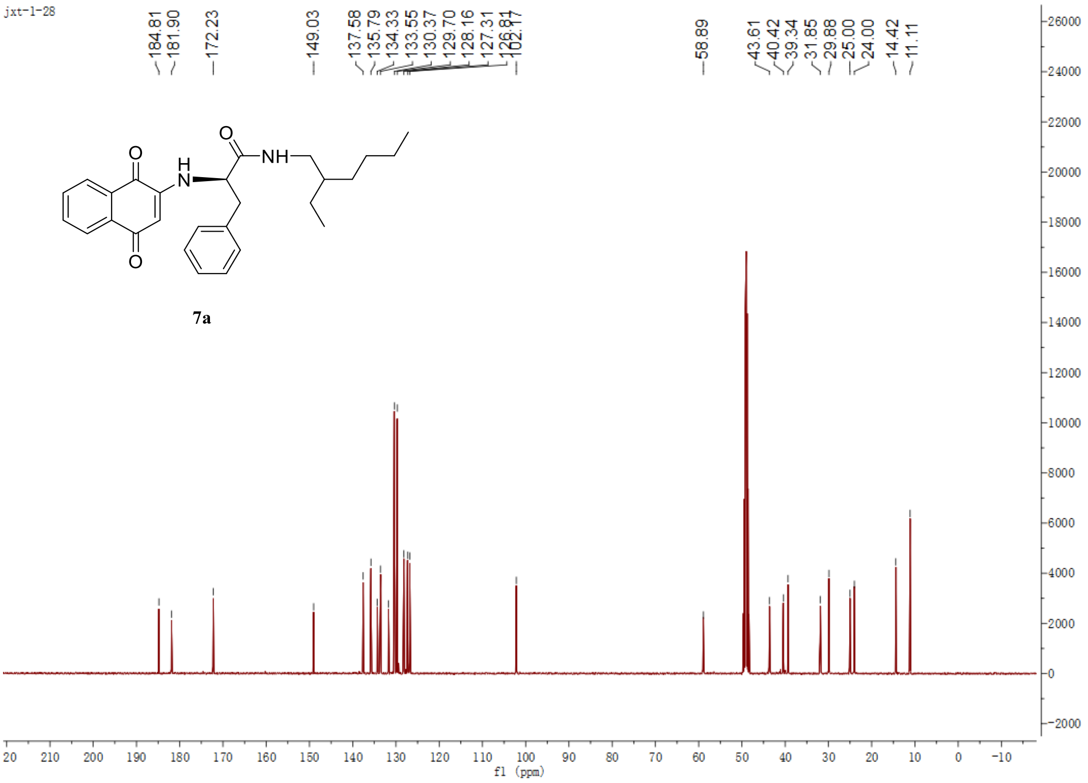

Figure S2. ^{13}C NMR Spectrum of compound **7a**.

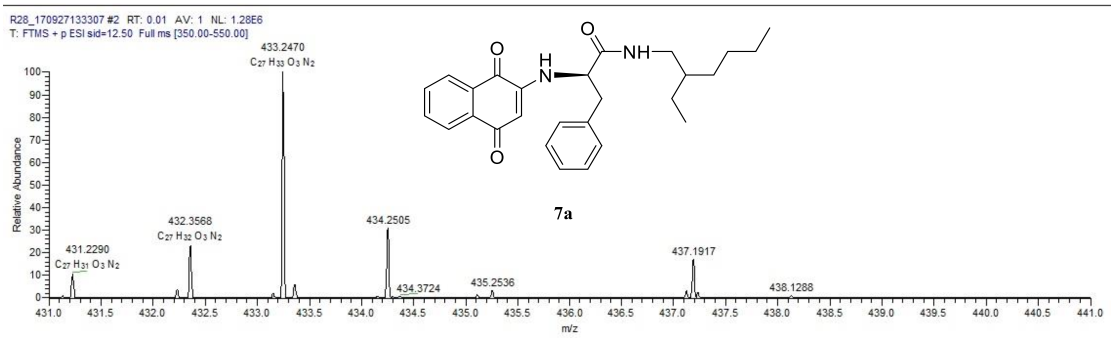

Figure S3. HRMS Spectrum of compound **7a**.

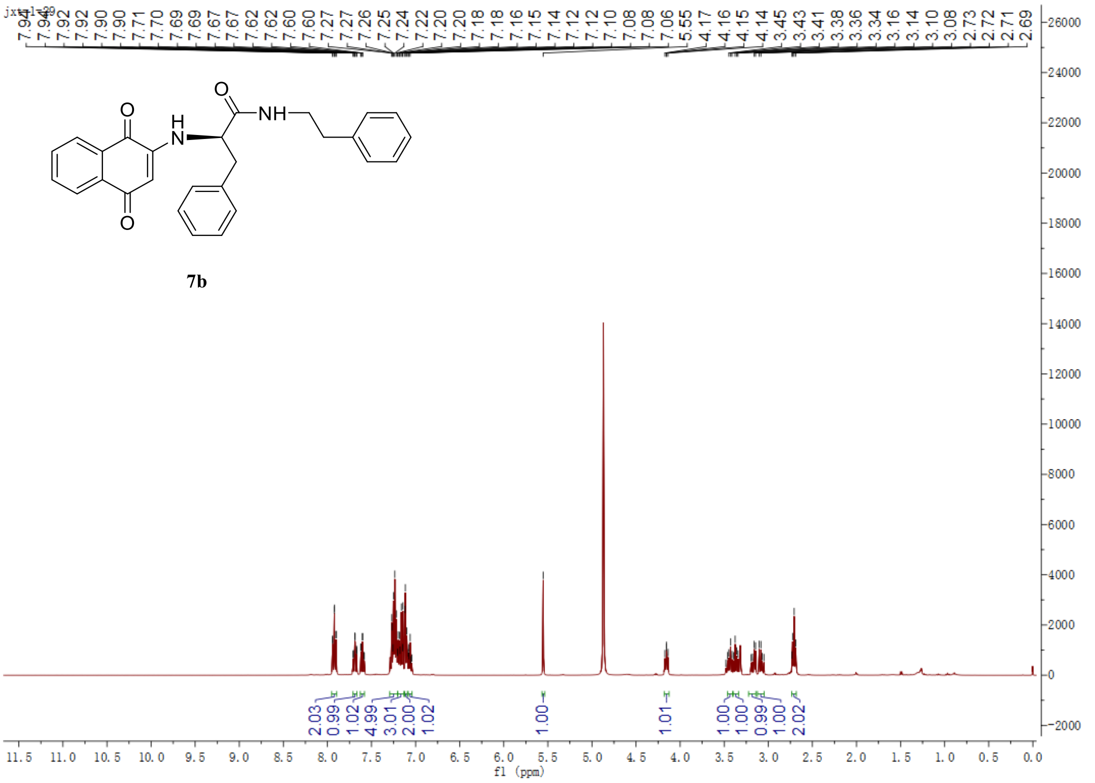

Figure S4. ^{1}H NMR Spectrum of compound **7b**.

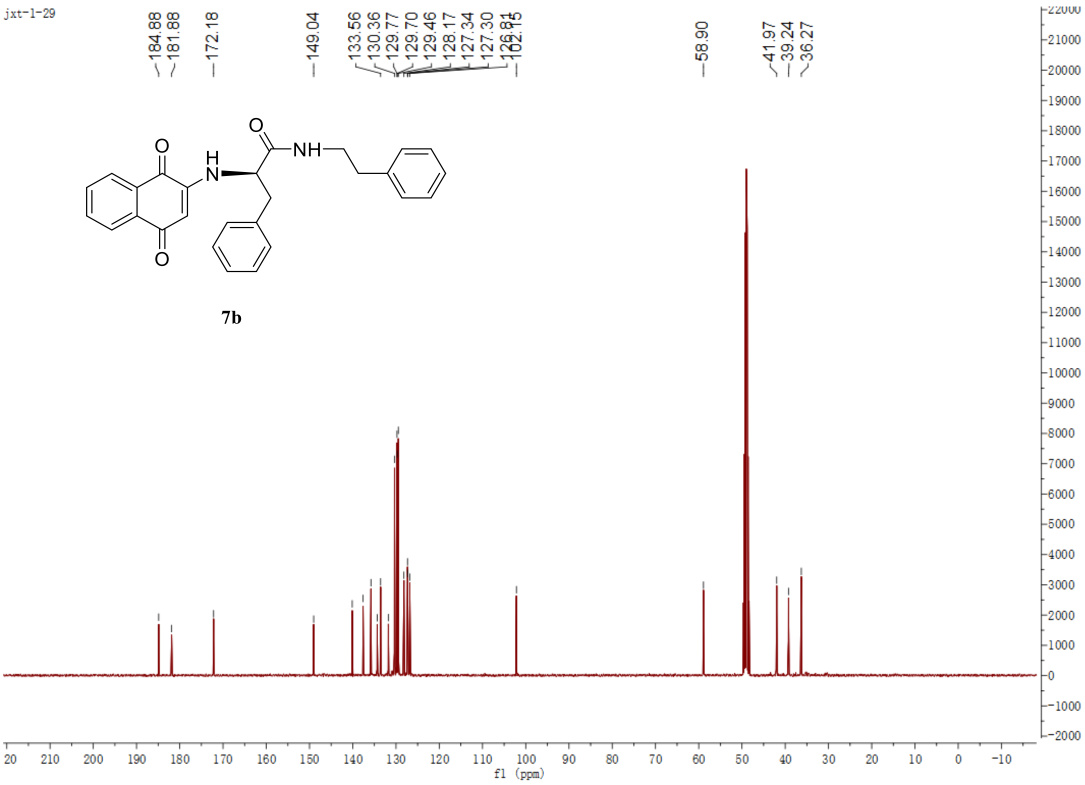

Figure S5. ^{13}C NMR Spectrum of compound **7b**.

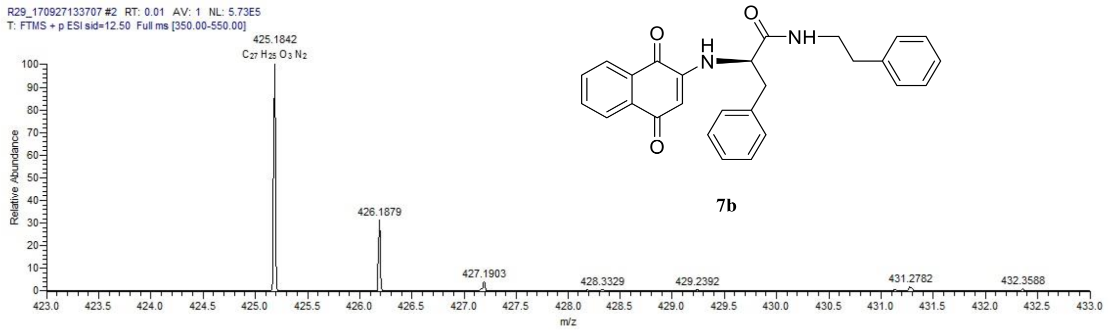

Figure S6. HRMS Spectrum of compound **7b**.

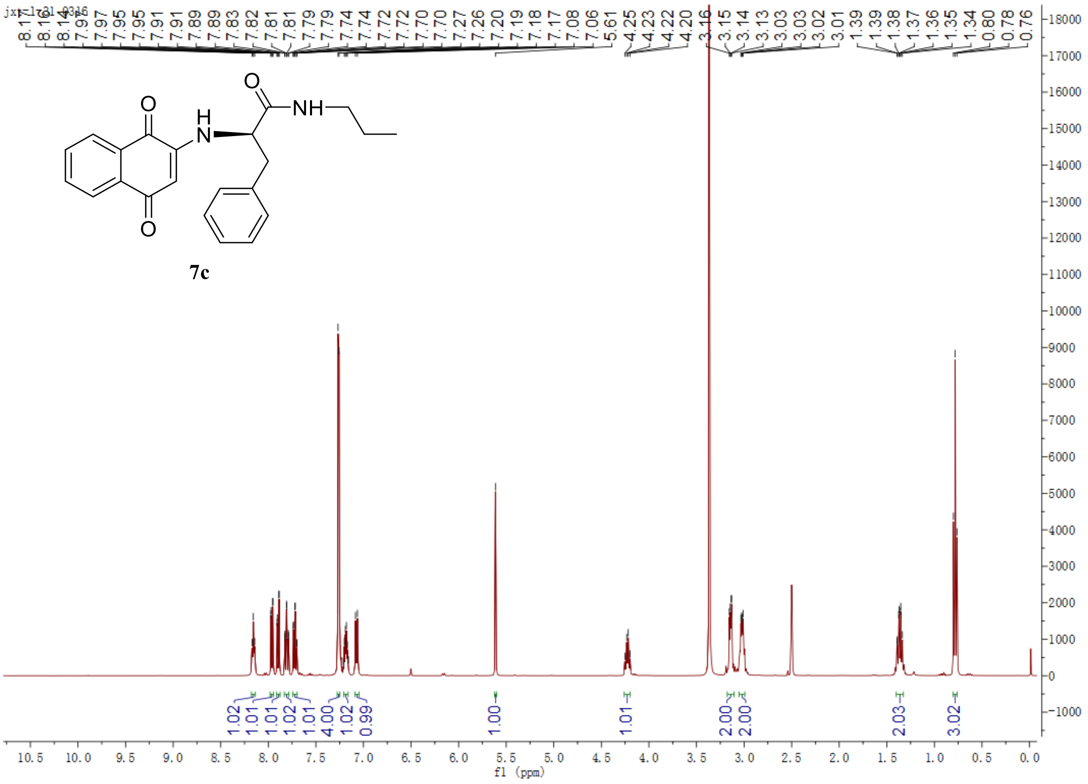

Figure S7. ^{1}H NMR Spectrum of compound **7c**.

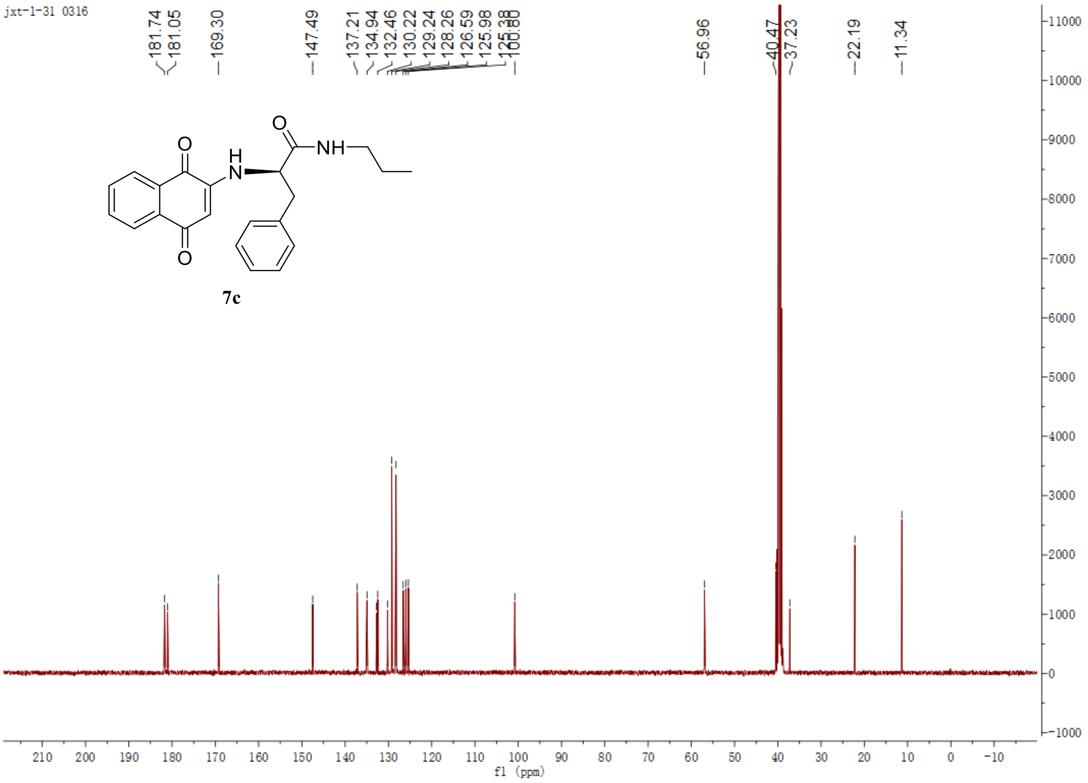

Figure S8. ^{13}C NMR Spectrum of compound **7c**.

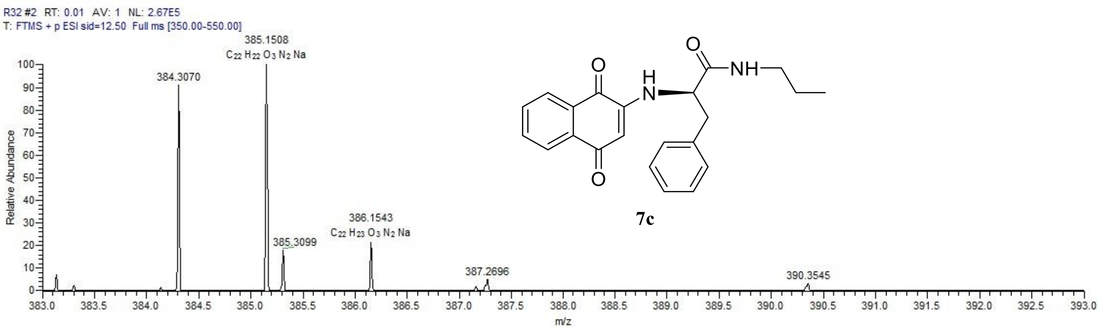

Figure S9. HRMS Spectrum of compound **7c**.

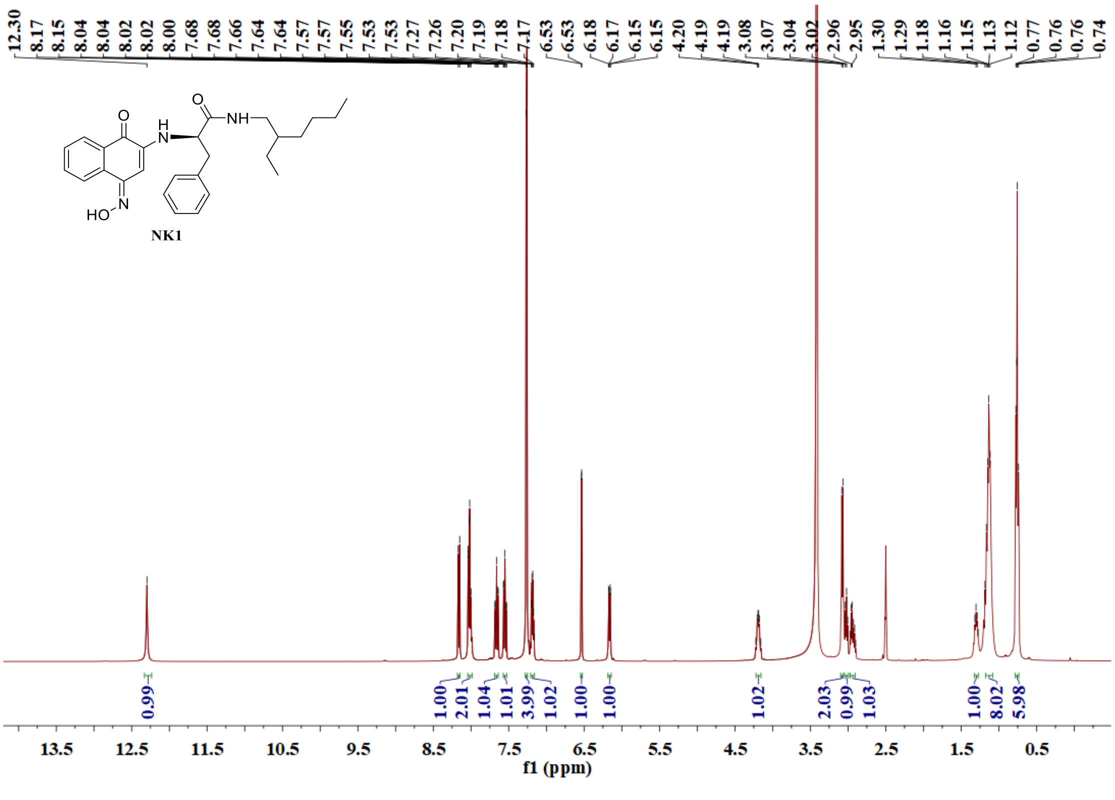

Figure S10. ^{1}H NMR Spectrum of compound **NK1**.

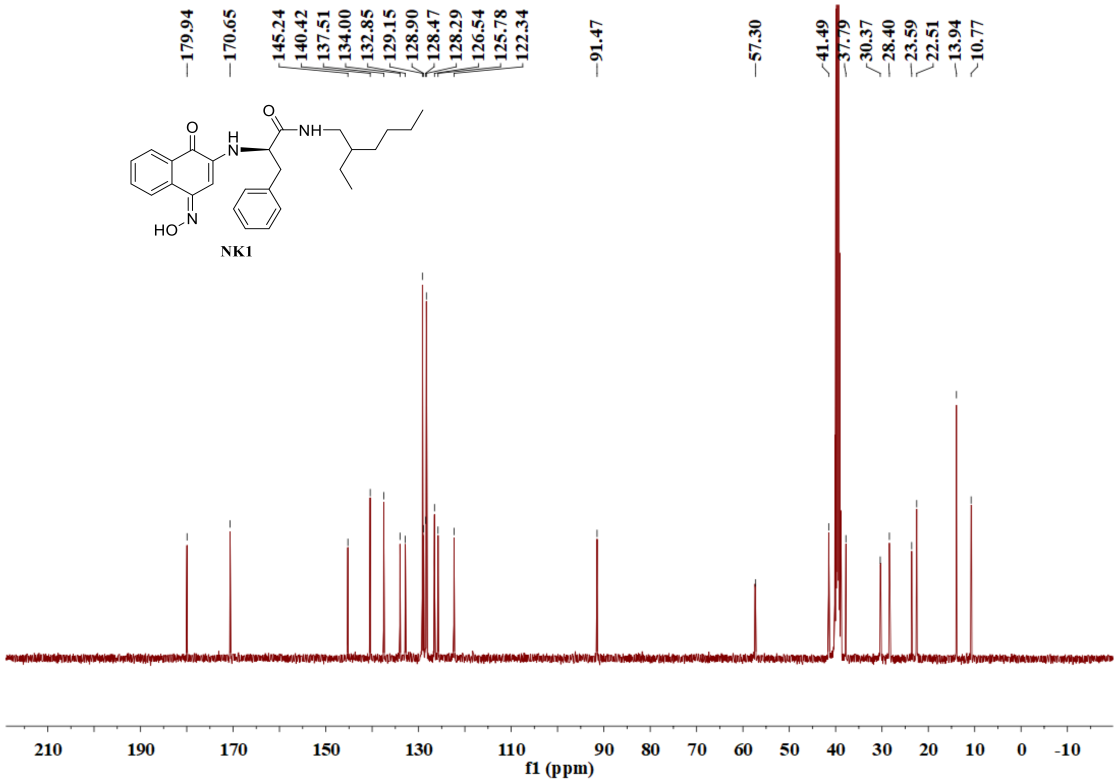

Figure S11. ^{13}C NMR Spectrum of compound **NK1**.

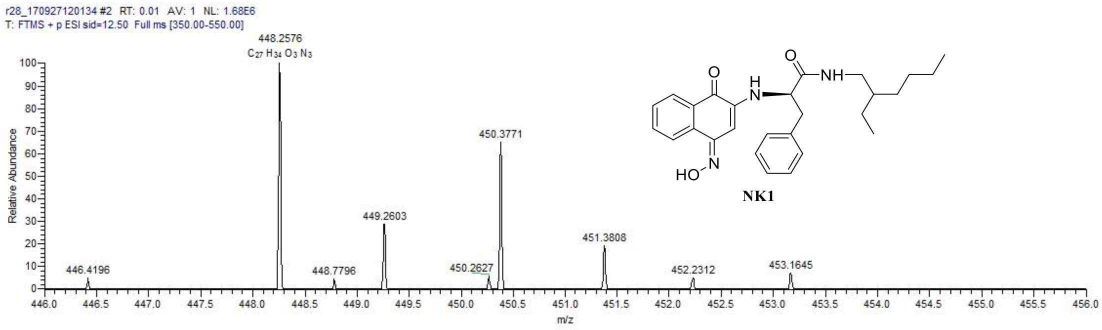

Figure S12. HRMS Spectrum of compound **NK1**.

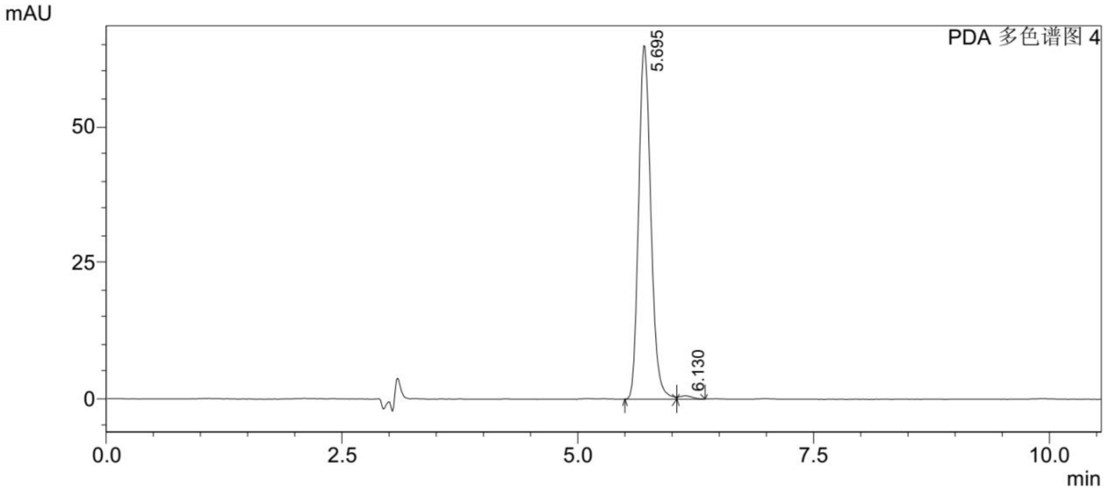

Figure S13. HPLC Spectrum of compound **NK1**.

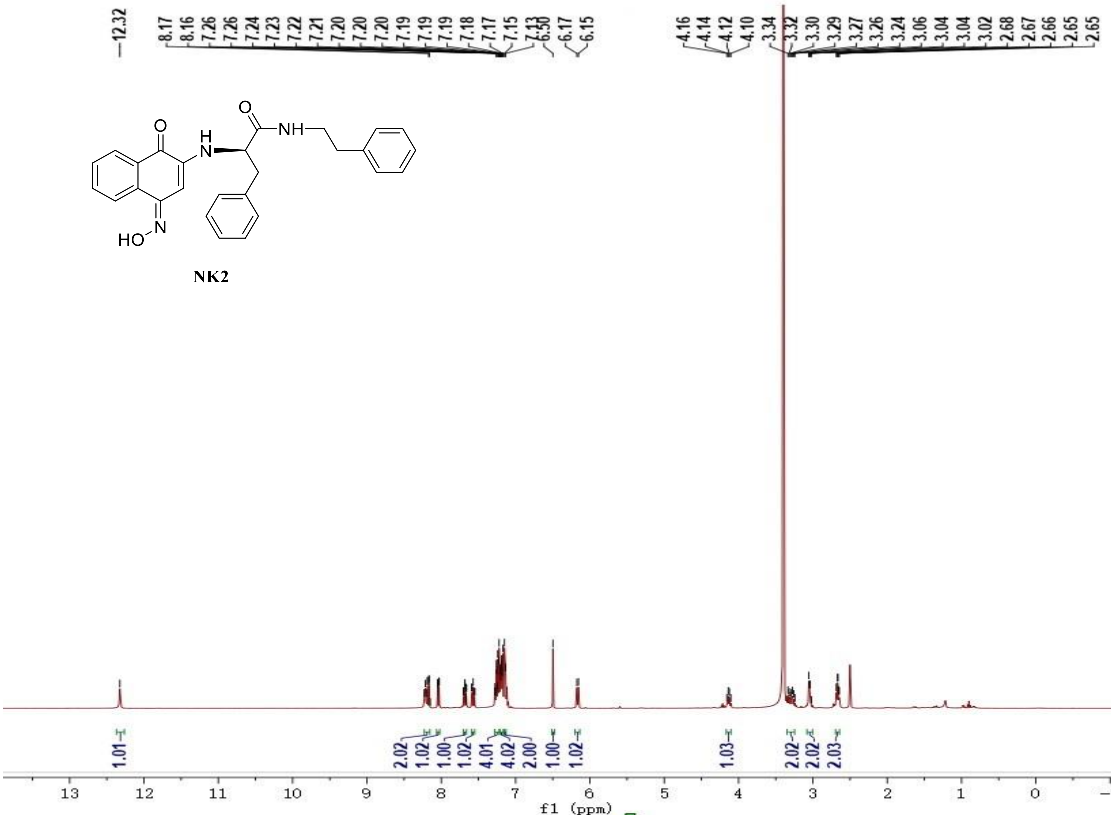

Figure S14. ^{1}H NMR Spectrum of compound **NK2**.

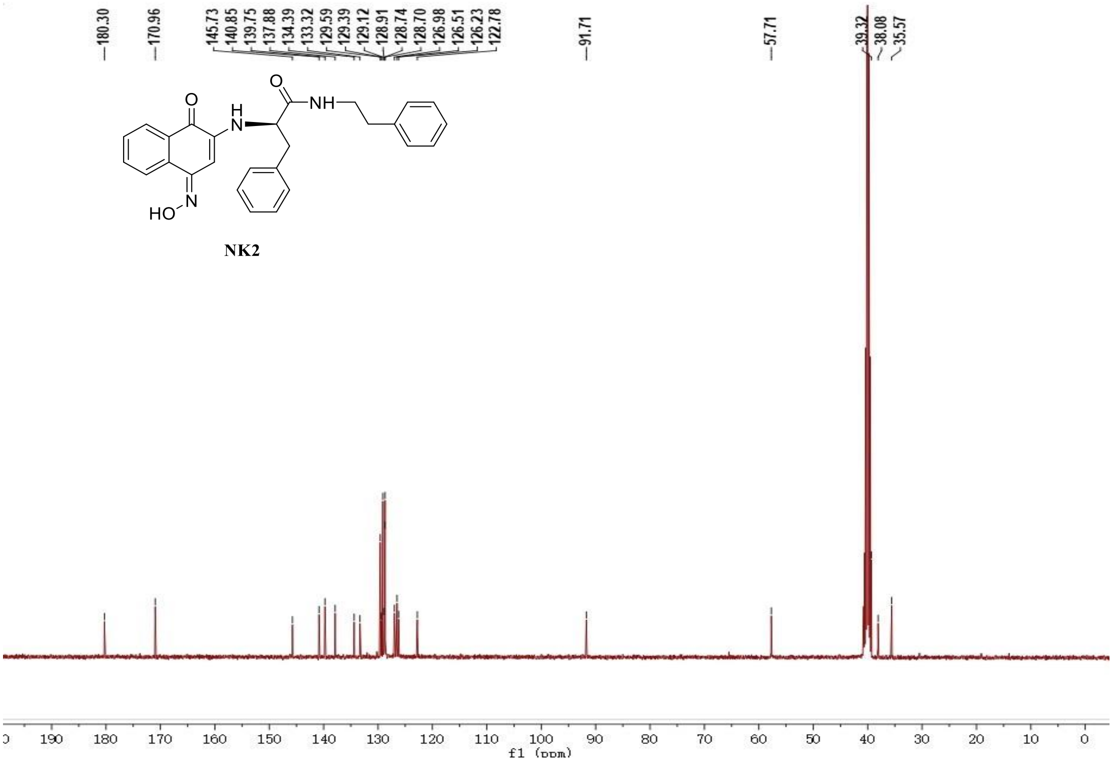

Figure S15. ^{13}C NMR Spectrum of compound **NK2**.

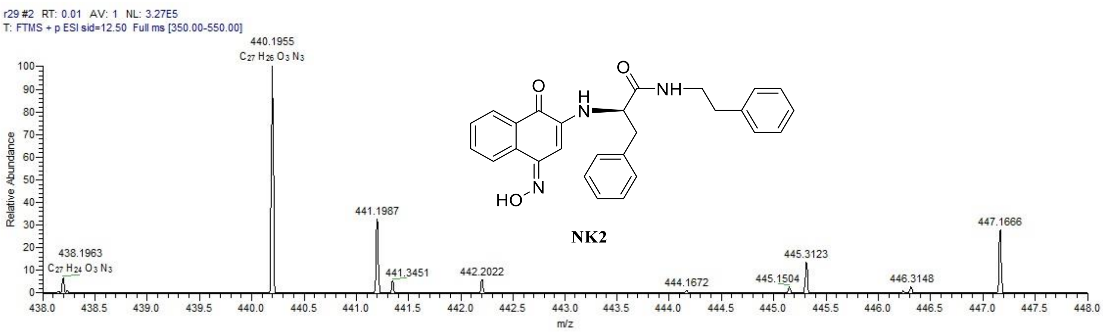

Figure S16. HRMS Spectrum of compound **NK2**.

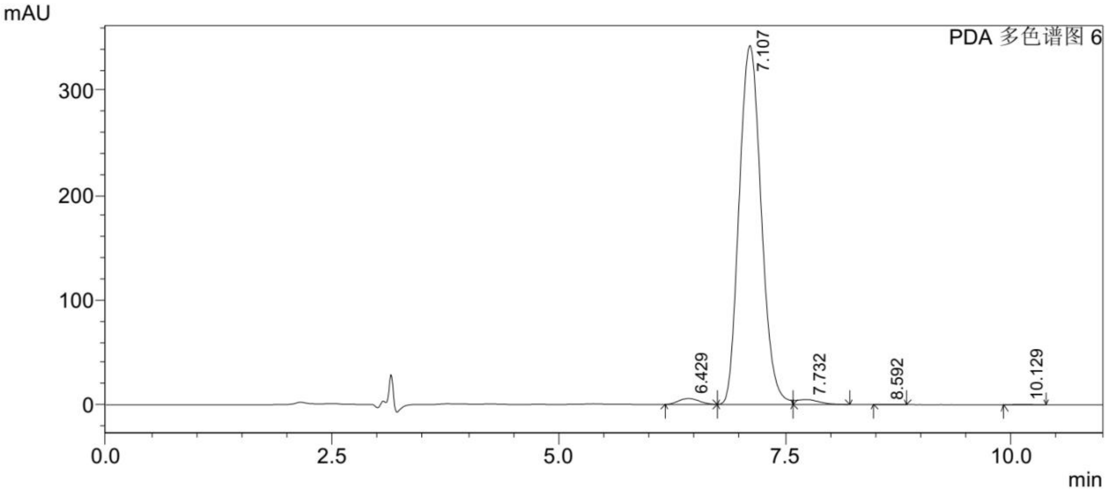

Figure S17. HPLC Spectrum of compound **NK2**.

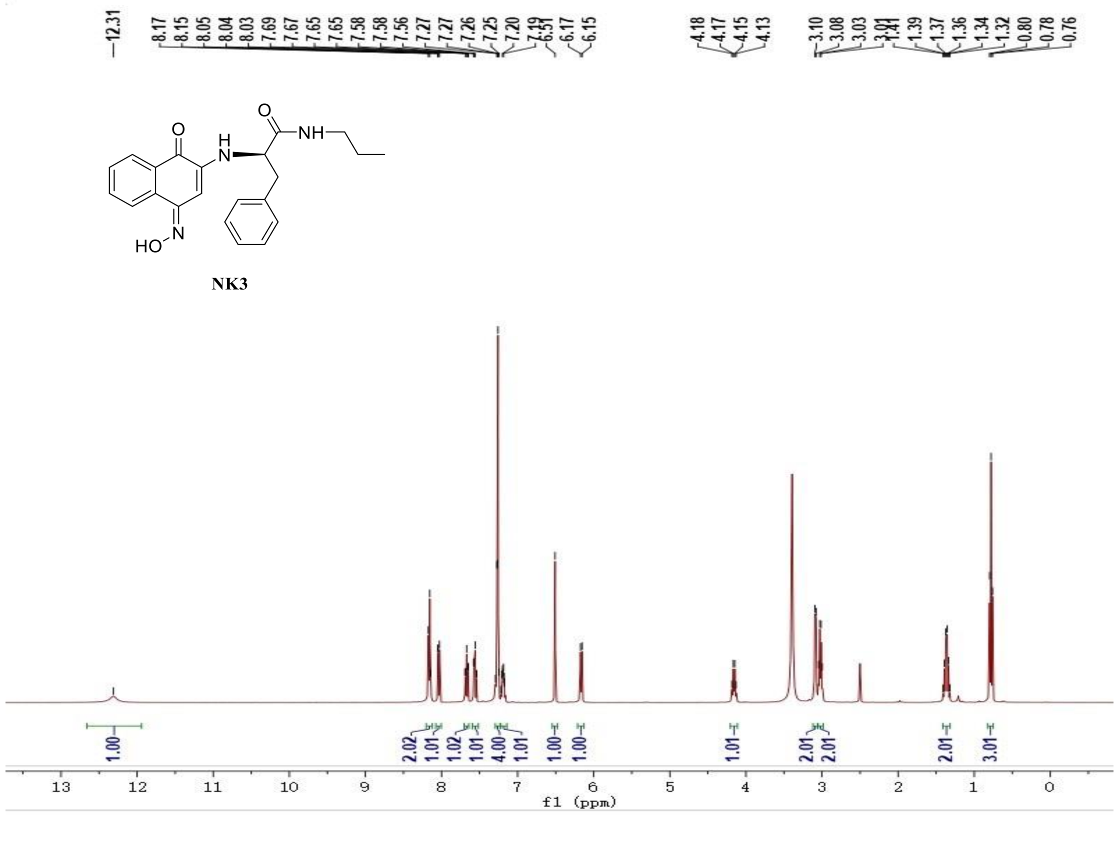

Figure S18. ^{1}H NMR Spectrum of compound **NK3**.

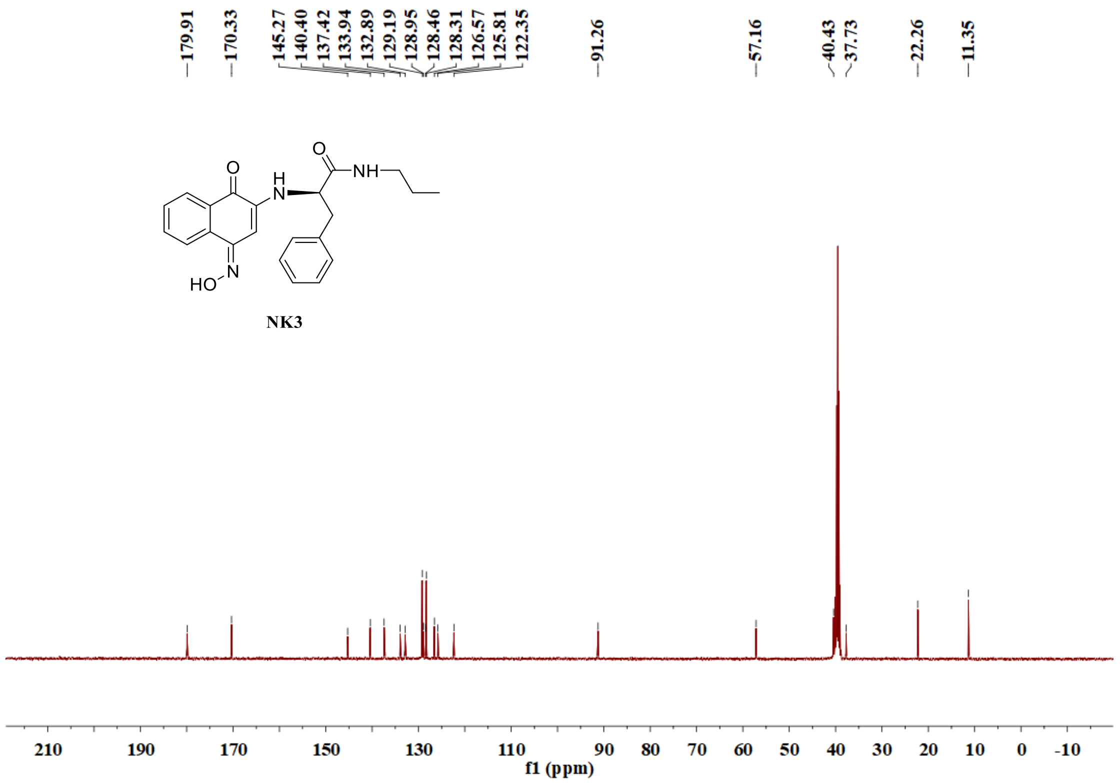

Figure S19. ^{13}C NMR Spectrum of compound **NK3**.

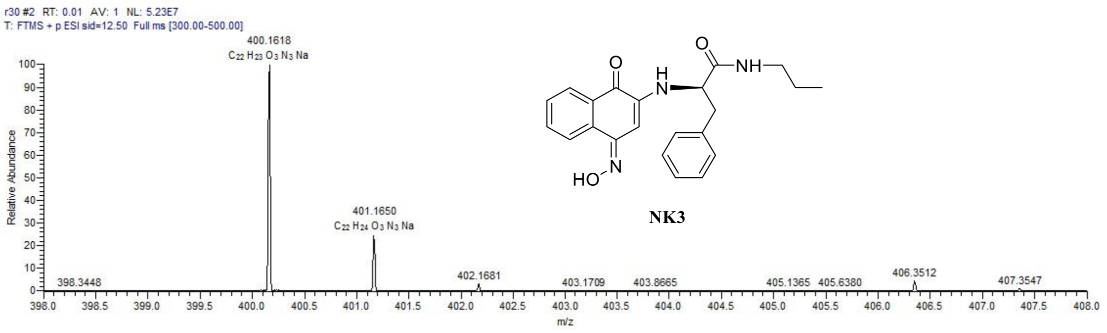

Figure S20. HRMS Spectrum of compound **NK3**.

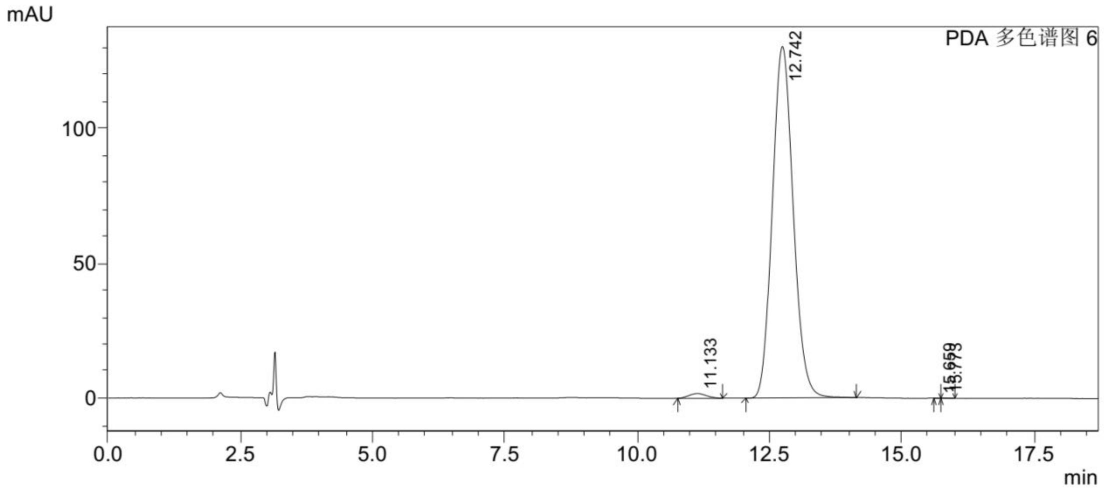

Figure S21. HPLC Spectrum of compound **NK3**.
